# Influence of Environmental Pollution and Living Conditions on Parasite Transmission among Indigenous Ecuadorians

**DOI:** 10.3390/ijerph19116901

**Published:** 2022-06-04

**Authors:** Luisa Carolina González-Ramírez, Ximena Robalino-Flores, Eliana De la Torre, Paúl Parra-Mayorga, José Gregorio Prato, María Trelis, Màrius Vicent Fuentes

**Affiliations:** 1 Grupo de Investigación “Análisis de Muestras Biológicas y Forenses”, Laboratorio Clínico, Facultad de Ciencias de la Salud, Universidad Nacional de Chimborazo (UNACH), Av. Antonio José de Sucre, Riobamba 060103, Ecuador; xrobalino@unach.edu.ec (X.R.-F.); edelatorre@unach.edu.ec (E.D.l.T.); 2 Grupo de Investigación “Análisis de Muestras Biológicas y Forenses”, Carrera de Comunicación, Facultad de Ciencias Políticas y Administrativas, Universidad Nacional de Chimborazo (UNACH), Riobamba 060103, Ecuador; pparra@unach.edu.ec; 3 Grupo de Investigación Estudios Interdisciplinarios, Facultad de Ingeniería, Universidad Nacional de Chimborazo (UNACH), Riobamba 060103, Ecuador; 4 Unidad Común de Investigación en Endocrinología, Nutrición y Dietética Clínica, Instituto de Investigaciones Sanitarias La Fe (IISLAFE), Universitat de València, Av. Fernando Abril Martorell, 106, 46026 Valencia, Spain; maria.trelis@uv.es; 5 Grupo de Investigación “Parásitos y Salud”, Departament de Farmàcia i Tecnologia Farmacèutica i Parasitologia, Facultat de Farmàcia, Universitat de València, Burjassot, 46010 Valencia, Spain; mario.v.fuentes@uv.es

**Keywords:** environmental contamination, intestinal parasites, vehicles, vectors, reservoirs

## Abstract

The purpose of this study was to evaluate the influence of environmental pollution and the living conditions of indigenous Ecuadorians on the transmission of enteroparasites in an Andean agricultural area located at high altitude. Environmental pollution was recorded after observation in each community. The parasites were identified by microscopic sediment analysis using physiological saline solution from macerated arthropods, washed vegetables, and human stools, utilizing four coproparasitological techniques (direct examination, Kato–Katz, ether concentration, and Ziehl–Neelsen). The results show that the inadequate disposal of human and animal excreta that contaminate soil and water, incorrect food hygiene, inadequate sanitary infrastructure in houses, a lack of animal veterinary care, and rodent proliferation are important reservoirs of zoonotic parasites. The use of excrement as fertilizer increases the number of flies, which act as mechanical vectors, and vegetables grown in areas with disperse infective parasitic forms act as vehicles that are marketed at the local, regional, and international levels. These analyses verify contamination levels of 52.7% in mechanical vectors, 70.6% in vegetables, and 98.2% in human stools. The agricultural communities analyzed maintained poor hygienic–sanitary and environmental conditions, which had a significant influence on the transmission of enteroparasites that affect human health.

## 1. Introduction

Enteric parasites are a public health problem in developing countries [1], and are considered an indicator of low educational and health levels in a community, making it necessary to provide an emergency response to minimize their transmission.

According to UNICEF-OMS records [2], 10% of the world’s population consumes food irrigated with wastewater, and 32% do not have access to basic sanitation services, which contributes to the transmission of enteroparasites—a phenomenon related to delayed growth and delayed psychomotor and cognitive development in children [3]. Among these parasites, *Entamoeba histolytica*, *Giardia duodenalis*, *Cyclospora cayetanensis*, *Cryptosporidium* spp., *Balantidium coli*, *Taenia solium* (cysticercosis), *Ascaris lumbricoides*, *Trichuris trichiura* and *Hymenolepis nana* are of particular note, due to their pathogenesis and their prevalence [4]. Their infective forms can be found as result of fecal contamination, great resistance to environmental conditions, survival in soil and water for domestic use, presence in non-treated water, presence in vegetables that are eaten raw, and food handled with poor hygiene [5,6].

Ecuador is among the countries with the highest prevalence of intestinal parasites: 78.3% in schoolchildren of Sinincay parish (Azuay Province) [7], 45.3% in schoolchildren of Paján Canton [8], 30.6% in Jipijapa Canton (Manabí Province) [9], and 35.1% in the town of Riobamba (Chimborazo Province) [10].

The inhabitants of the rural areas of the Ecuadorian Andes maintain a low educational level [11], and are unaware of the hygienic–sanitary measures that should be practiced when carrying out agricultural activities, increasing the risk of the contamination of agricultural products, which then become vehicles of enteroparasites when they are marketed at a regional, national, or international level [12,13]. In rural indigenous populations, the prevalence of intestinal parasites is higher than in urban environments. The Tsáchilas indigenous people, who reside west of the Andean Mountains, reach levels of parasitism as high as 68% [14]. This prevalence increases in high-altitude Andean areas that maintain conditions of extreme poverty, as is the case for Quichua children (Chimborazo Province), with 85.7% enteroparasite prevalence [15].

In the mountainous region of Chimborazo, intestinal parasites are a public health issue; infected individuals remain undiagnosed and untreated, and may suffer irreversible health changes [4]. In addition, epidemiological studies are necessary in order to elucidate the sources of infection, some of which are unknown. The risk factors associated with enteroparasite transmission must be identified when applying an integrated control program that prevents immediate reinfection after pharmacological treatment [16].

Within this context, the aim of the present study was to identify the influence of environmental contamination and living conditions as possible risk factors associated with the intestinal transmission of parasites among the indigenous population of the San Andres parish in the province of Chimborazo. The findings of this study will fill vital gaps and provide beneficial insights and information on the epidemiology and dynamics of parasitic infections and their associated factors. Such data will be valuable for public health authorities to justify and facilitate the reassessment of existing control measures to reduce the prevalence of parasitic infections in these communities.

## 2. Materials and Methods

### 2.1. Study Area

**Figure 1:**
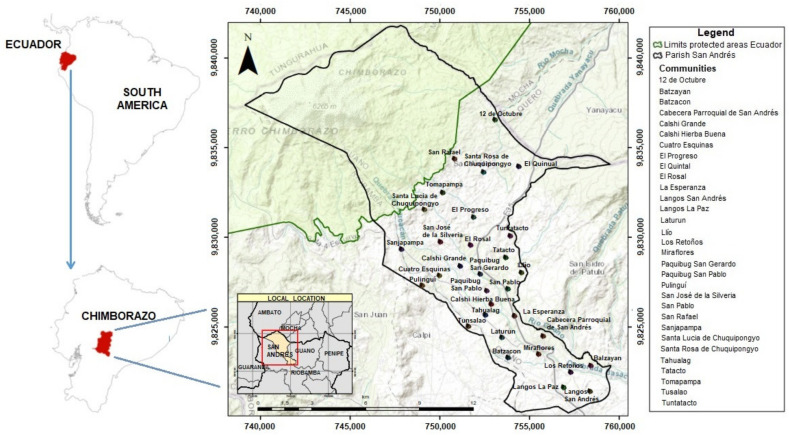
Geographical locations of the studied communities.

The study area was the parish of San Andres, Guano Canton, located northwest of Chimborazo Province (Ecuador), with a total of 13,481 habitants across 34 communities that cover a territorial extension of 159.9 km^2^. The altitude is between 3020 and 6310 m above sea level, with temperatures ranging from 0 to 19 °C, with an average of 11.2 °C, and rainfall that varies between 250 and 500 mm/year, with agricultural activity being the main economic means of subsistence [11]. The representative sample (*n*) was taken from 31 communities (Figure 1).

The six risk factors that predispose the transmission of enteroparasites in the San Andres communities are described below.

#### 2.1.1. Fecal Soil Contamination

Fecal soil contamination occurs when animals are parasitized (without veterinary control) and move freely in the fields, fertilizing cultures with fresh animal stools.

**Figure 2:**
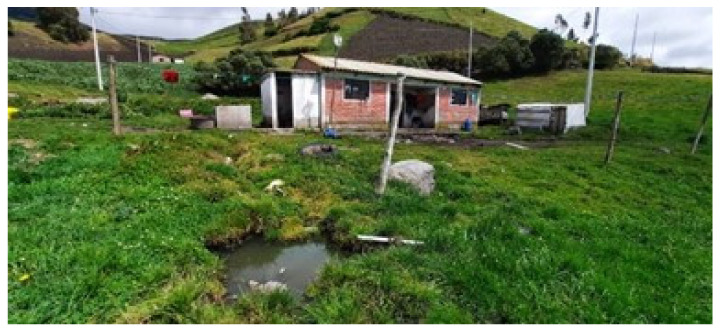
House with a leaky septic tank underground.

Human defecation in open air is observed, even though the majority of the population has a toilet in their house. In addition, the sewage is discarded in inadequately built septic tanks, and often leaks underground and, in some cases, overflows in the surrounding areas close to the houses (Figure 2).

#### 2.1.2. Fecal Water Contamination

**Figure 3:**
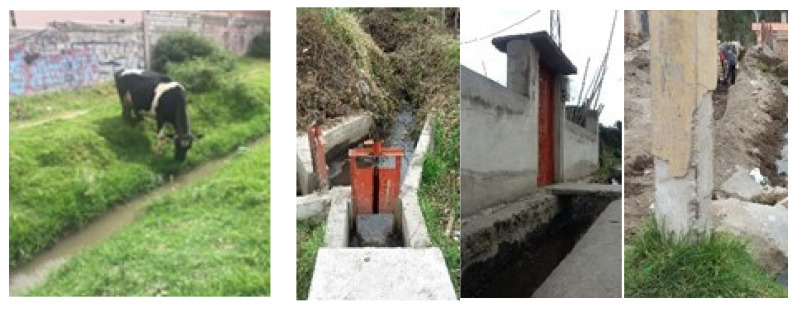
Irrigation channels.

The hydric dispersion of enteroparasites occurs due to the fecal contamination of the water that flows through irrigation canals (Figure 3), when the runoff from the rain drags the excrement from the ground drains on its way. In addition to this, the artificial wells maintained by livestock producers to supply their animals are also contaminated.

Deficiencies are also observed in water consumption. Although the houses have tanks and distribution pipes, the chlorination of the communities does not meet the national standard, stipulating concentrations between 0.3 and 1.5 ppm of residual chlorine [17], which translates into insufficient or excessive amounts of chlorine [18].

#### 2.1.3. Pest Control

In rural areas there are no pest control programs. Flies abound in all communities, and some spiders and beetles are found; there are no other arthropods of recognized importance.

#### 2.1.4. Food Hygiene

**Figure 4:**
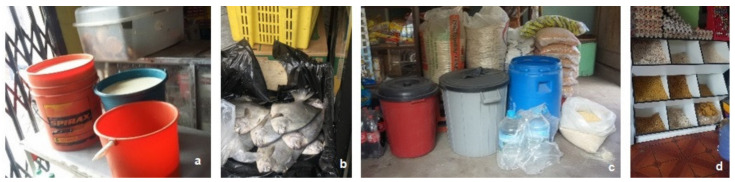
(**a**) Milk, (**b**) fish without refrigeration, (**c**) grains and cereals, and (**d**) pasta dispensed without adequate hygiene.

Food is sold in stores or on the street with poor hygiene, being kept without protection; pasta, grains, and cereals are stored in open containers and bags, where they are easily reached by insects and rats, and dairy and meat products are unrefrigerated (Figure 4). In no communities was there sanitary control by vendors, and inadequate hygiene was observed in the handling of food.

Additionally, the consumption of chochos (*Lupinus mutabilis*) is considered risky; chochos are debittered in the polluted water bodies of the region, and although they are cooked, they are handled unhygienically in street stalls, and are eaten without hygiene treatment before consumption.

#### 2.1.5. Contact with Animals

**Figure 5:**
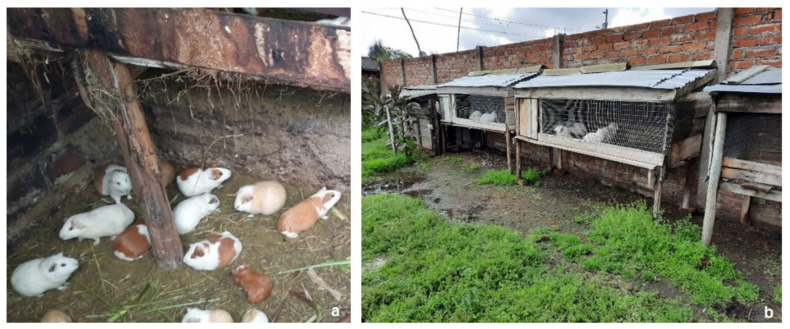
(**a**) Guinea pigs raised inside the house; (**b**) rabbits in aerial cages.

Community residents are in close contact with pets, livestock, and farm animals, which remain domicile or peridomicile (Figure 5). The significant number of stray dogs constitutes an epidemiological risk in the communities, due to the fact that they spread their excrement in places of recreation, mainly used by children.

In most houses, guinea pigs (*Cavia porcellus*) are raised, and are consumed and marketed as an important indigenous culinary tradition of the Ecuadorian Andes. These rodents are raised in houses or in aerial cages together with rabbits, to collect their droppings for use as fertilizer.

Around the houses, various veterinary species act as reservoirs of intestinal parasites, such as cows, sheep, goats, llamas, horses, donkeys, pigs, chickens, ducks, geese, pigeons, etc., and are all in frequent contact with their breeders [19].

#### 2.1.6. Housing Conditions

**Figure 6:**
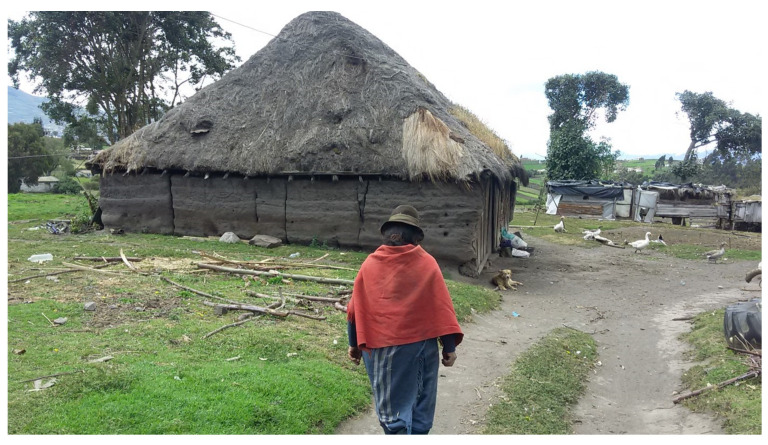
Shacks with poor hygienic conditions.

The houses near the main pathways have adequate sanitary infrastructure, electricity, and water services. However, among the houses located at higher altitudes are shacks with mud walls, dirt floors and thatched roofs, which do not guarantee basic sanitation services for their inhabitants (Figure 6).

### 2.2. Registry of Environmental Indicators

**Figure 7:**
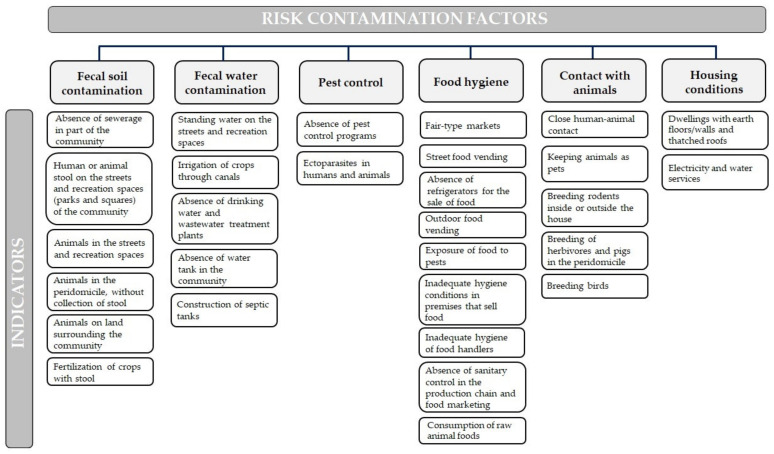
Risk contamination factors of infection in communities of San Andrés.

Based on the WHO and PAHO criteria to assess environmental risks to human health related to poverty and underdevelopment [20], 29 indicators were selected among the 6 risk factors for parasite transmission considered (Figure 7). To evaluate these indicators, each of the 31 communities selected in the sampling was visited.

### 2.3. Sampling

This section indicates how the samples of communities, arthropods, fruits and vegetables, and human residents of each community were calculated.

#### 2.3.1. Communities Sample

The representative sample (*n*) was taken from 31 communities (Figure 1), and was determined by applying Equation (1):

(1) $$n=\frac{Z^{2}*\;p\;*\;q\;*\;N}{\left(e^{2}*\;\left(N-1\right)\right)+Z^{2}*\;p\;*\;q\;}$$

where *N* represents the size of the population (*N* = 34), *Z* is the value at the reliability level or significance level (for 95% reliability level, *Z* = 1.96 [21]), *e* is the acceptable error of the sample size, *p* is the population proportion (*p* = 0.75), and *q* is 1 − *p*.

The 31 communities were randomly selected using Microsoft Excel version 16.

It is important to clarify that it was only possible to carry out the coprological analysis in people who lived in six communities, because the collection of stool samples was carried out in each house, far in the mountains, and the indigenous people are often unwilling to collaborate, requiring several visits.

#### 2.3.2. Arthropods, Fruits and Vegetables Sample

Due to the fact that the population of arthropods and fruit and vegetables is infinite, the sample was calculated by applying Cochran’s formula [21], as shown in Equation (2):

(2) $$n=\frac{Z^{2}*\;p\;*\;q\;}{\;e^{2}\;}$$

where *Z* is the value at the significance level (for 95% reliability level, *Z* = 1.96), *e* is the desired level of precision (*e* = 0.05), *p* is the population proportion (*p* = 0.25), and *q* is 1 − *p*.

#### 2.3.3. Human Sample 

The study sample size of the residents of the communities in the San Andres parish was calculated according to information from the National Institute of Statistics of Ecuador (INEC) [22], applying Equation (3):

(3) $$n=\frac{Z^{2}*\;S^{2}}{e^{2}+\frac{Z^{2}*\;S^{2}}{N}}$$

where *Z* = 1.96, *S*^2^ is the variance, *e* is the acceptable error of the sample size, and *N* is the size of the population.

The people included in the sample were selected based on the population size projected by the INEC for San Andrés parish; as a result, Community 1 had a population of 1052, and a sample of 109; Community 2 had a population of 590, and a sample of 62; Community 3 had a population of 506, and a sample of 54; Community 4 had a population of 205, and a sample of 22; Community 5 had a population of 61, and a sample of 7; and Community 6 had a population of 194, and a sample of 21.

### 2.4. Collection of Environmental Pollution Information

A structured checklist (Figure 7) was used to record the environmental conditions as risk factors associated with parasitic transmission. The data were collected through Google Forms and exported to Microsoft Excel version 16.

### 2.5. Processing and Analysis Samples

For the determination of the parasite contamination of arthropods and vegetables, as well as parasitic infection in humans, the following were collected: 300 arthropods, including 186 flies, 71 spiders, and 43 beetles; 320 agricultural products, including 146 fruits (48 strawberries, 33 blackberries, 6 Peruvian groundcherries, 19 tomatoes, 14 apples, 17 guavas, and 9 lemons) and 174 vegetables (27 potatoes, 23 carrots, 8 beets, 36 radishes, 16 white onions, 16 red onions, 6 broccoli, 8 lettuce, 11 celery, 7 parsley, 6 coriander, and 10 chochos); and 396 human stool samples of Quichua indigenous people (241 female, 155 male; age range 4–91 years) from 6 communities of the San Andres parish.

#### 2.5.1. Arthropod Processing and Analysis

For parasite detection, each fly, spider, and beetle captured was submerged and macerated in 100 µL of physiological saline solution in an Eppendorf tube. After allowing spontaneous sedimentation for 1 h, 10 µL of each sediment was analyzed under a light microscope, using 10× and 40× objectives. A drop of the sediment was stained using the Ziehl–Neelsen technique [23], and visualized using a 100× objective, using the micrometer ocular to differentiate *Cryptosporidium* from *Cyclospora*.

#### 2.5.2. Vegetable Product Processing and Analysis

For each type of vegetable, 200 g was weighed and individually placed in sterile plastic containers with smooth walls, and 300 mL of sterile saline solution was added to each sample, which after being closed hermetically were shaken vigorously and kept at rest for 1 h before performing the direct examination of the sediment with a magnification of 100× and 400×, using Lugol’s iodine. A sediment drop was stained with Ziehl–Neelsen reagent [23], and the ocular micrometer was used to identify the parasites according to their size.

#### 2.5.3. Human Stool Sample Processing and Analysis

Fresh stool samples were processed using four complementary coproparasitological techniques: direct examination with physiological saline solutions and Lugol’s iodine; a Kato–Katz slide made from each stool sample following WHO recommendations, using a template delivering about 41.7 mg of stool [24]; formol–ether concentration [25]; and one aliquot of sediment obtained via this technique, stained using the modified Ziehl–Neelsen method [23]. Finally, the microscopic observation was carried out using objectives of 10×, 40×, and 100×, and the ocular micrometer when necessary.

### 2.6. Ethical Considerations

Ethical approval for the study was provided by the Ethics Committee of the Universidad Central del Ecuador (license number 0004-EXT-2021). To each person, we explained the aim and objectives of the study, and each signed informed consent and assent (in cases of underage people) to participate in this research.

### 2.7. Statistical Analysis

Statistical analysis was performed with SPSS software version 24 (Chicago, IL, USA). Probability values were considered to be statistically significant when the calculated *p*-value was equal to or less than 0.05. The differences in parasitic contamination among the different categories (communities; enteroparasites and risk factors; flies and spiders; fruits and vegetables; protozoa and helminths; parasitic species; correlation between carriers) was compared using Pearson’s chi-squared test (*χ*^2^) and Fisher’s exact test (EF), where appropriate.

To determine the significant associations between the different risk factors in the six communities, odds ratios (ORs) were determined, and we measured the effect of arthropods as a mechanical vector and of food as a vehicle for parasites; the point estimates and 95% confidence interval (95% CI) estimates were used to assess the differences in proportions in independent samples, as well as the point estimate relative risk (RR).

For the analysis of the concordance diagrams between parasites and carriers (i.e., arthropods, food, and humans), the Cohen’s kappa statistical method was used (coefficient values < 0 as indicating no agreement, and 0–0.20 as slight, 0.21–0.40 as fair, 0.41–0.60 as moderate, 0.61–0.80 as substantial, and 0.81–1 as almost perfect agreement) [26].

## 3. Results

This section shows the results regarding environmental pollution and the living conditions of the inhabitants of San Andres. Furthermore, it shows the results in terms of parasitic contamination in arthropods, vegetable crops, and infected humans.

### 3.1. Risk Factors

**Figure 8:**
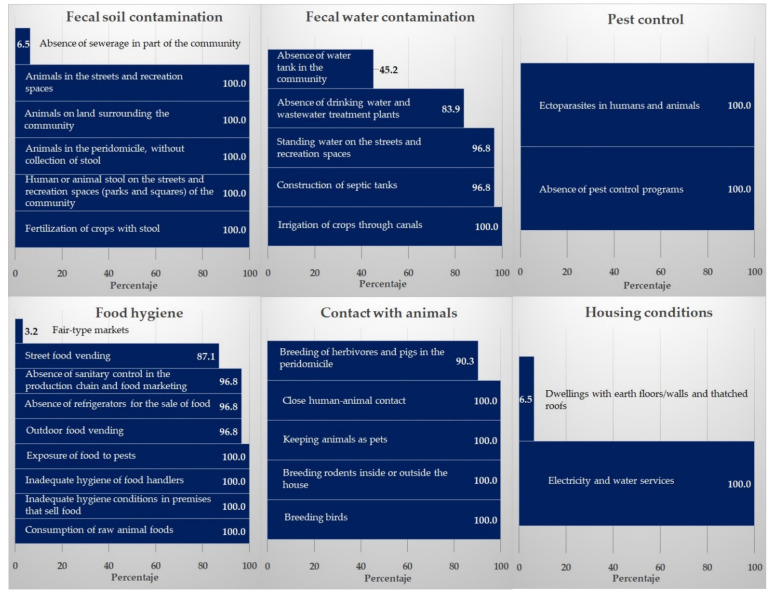
Risk factors associated with the transmission of intestinal parasites in San Andres.

Figure 8 shows the level of risk of parasitic contamination. The water was at a high level of risk in 29 of the 31 communities (93.5%); inadequate food hygiene was at a high risk level in 30 communities (96.8%); inadequate housing conditions were a risk factor in 2 communities (6.5%); and close contact with animals, fecal contamination of the soil, and the absence of pest control posed a high level of risk in all communities (100%).

When performing the data analysis, the average levels of the risk factors were determined: soil fecal contamination (0.84 ± 0.042) and water (0.85 ± 0.106); absence of pest control (1 ± 0); inadequate food hygiene (0.87 ± 0.055); contact with animals (0.98 ± 0.073); and inadequate housing conditions (0.53 ± 0.105), with an overall arithmetic mean of 0.85 ± 0.069. These results indicate that the risk variables have an influence of 85% as a transmission risk index, demonstrating the critical hygienic situation of the area.

The influence of the risk factors evaluated (i.e., environmental sanitation and habits) on the transmission of parasites in the six communities where the coprological study was carried out shows that it was possible to obtain a relationship between the enteroparasites and fecal water contamination (EF = 5.14; *p* = 0.038; OR = 11.5), absence of water tanks in the community (EF = 4.61; *p* ≤ 0.005; OR = 0.11), absence of sanitary control in the production chain and food marketing (EF = 11.9; *p* = 0.004; OR = 0.50), breeding of herbivores and pigs in the peridomicile (EF = 11.5; *p* = 0.004; OR = 0.52), and dwellings with earth floors/walls and thatched roofs (EF = 7.19; *p* = 0.032; OR = 0.67).

### 3.2. Parasitic Contamination in Arthropods, Vegetables, and Humans

The parasitic contamination of arthropods, vegetables, and humans is described below.

#### 3.2.1. Parasitic Contamination of Arthropods

Table 1 shows that the overall contamination of arthropods was 52.7%, and that no parasites were found in any of the beetles. The flies were found to be more contaminated than the spiders (χ^2^ = 98.659; *p* ≤ 0.0001), with the cysts of *Entamoeba* spp. (60.3%) being the most frequently detected parasite (χ^2^ = 821.261; *p* ≤ 0.0001). The protozoa were more frequent than the helminths (χ^2^ = 271.286; *p* ≤ 0.0001).

Table 1 also shows that the results were statistically significant when the 95% confidence intervals did not contain the zero value or the unit value. Therefore, the transmission of *Blastocystis* and *Entamoeba* was due more to the presence of flies than to that of other arthropods. Flies had a 6.3 times greater risk of carrying a protozoan than other arthropods and, in particular, the risk of carrying *Blastocystis* was 16 times greater for flies than for spiders. A similar phenomenon occurred with *Entamoeba*: the risk of transmission of this parasite was 9.5 times higher due to the presence of flies than to that of other arthropods.

#### 3.2.2. Parasitic Contamination of Agricultural Products (Fruit and Vegetables)

The overall contamination of agricultural products was 70.6%. Fruits (67.1%) and vegetables (73.6%) were equally contaminated, and the difference in percentage did not reach statistical significance. An exception was *Cyclospora*, the proportion of vegetables contaminated by this protozoan being greater than the proportion of fruits (95% CI: Ll = −0.2231, Ul = −0.0282). The vegetables had a 1573 times higher risk of being vehicles of *Cyclospora* than fruits (RR = 0.64), meaning that in vegetables *Cyclospora* transmission was 57.3% more risky than in fruits. In addition, it was possible to verify greater contamination by helminths for vegetables than for fruits (χ^2^ = 271.286; *p* ≤ 0.0001) (Table 2).

#### 3.2.3. Parasite Frequency in Humans

The results of the coprological analysis of 396 individuals, residing in 6 communities of the San Andres parish, reveal that 98.2% (389/396) were infected. Statistical significance was not reached when the total results were compared between communities. Statistical analysis confirmed the higher frequency of protozoa (98.2%) than helminths (8.3%) (*χ^2^* = 642.852; *p* < 0.0001).

According to the overall results, the most prevalent parasite was *Blastocystis*, at 86.6% (343/396) (*χ^2^* = 2565.395; *p* < 0.0001), followed by *Endolimax nana* at 69.4% (275/296) and *Entamoeba coli* at 57.6% (228/396). It is important to highlight the rates reached by pathogenic parasites: *Entamoeba histolytica/E. dispar*, 31.3%; *Giardia duodenalis*, 10.1%; *Cyclospora cayetanensis*, 4.3%; and *Cryptosporidium* spp., 3.5%. When comparing the frequency between the overall helminths, the highest infection level was confirmed for *Ascaris lumbricoides*, at 4.3% (17/396) (*χ^2^* = 27.247; *p* < 0.0001), and *Hymenolepis nana*, at 3.8% (15/396). 

Only when comparing the frequencies of helminth species were statistically significant differences detected between two communities, due to the higher frequency of *A. lumbricoides* in Community 5 (15.7%) (*χ^2^* = 20.246; *p* = 0.0011) and *H. nana* in Community 3 (12.3%) (*χ^2^* = 19.869; *p* = 0.0013) (Table 3).

#### 3.2.4. Parasite Correlations and Concordance between Variables (Humans, Vegetables, and Arthropods)

The correlation and concordance of parasites between the different variables (humans, vegetables, and arthropods) was determined according to the overall number and type of parasites.

##### Correlation and Concordance of Parasite Frequency between Vegetables and Humans According to Parasite Group

The comparative analysis of sarcodines (χ^2^ = 4.75; *p* = 0.3173), flagellates (χ^2^ = 0.014; *p* = 1), coccidia (χ^2^ = 0.018; *p* = 1), and chromists (χ^2^ = 1.10; *p* = 0.3068) between vegetables and humans showed that there were no significant differences between them. On the other hand, the sarcodines, flagellates, and coccidia found in vegetables and those diagnosed in humans were correlated.

**Figure 9:**
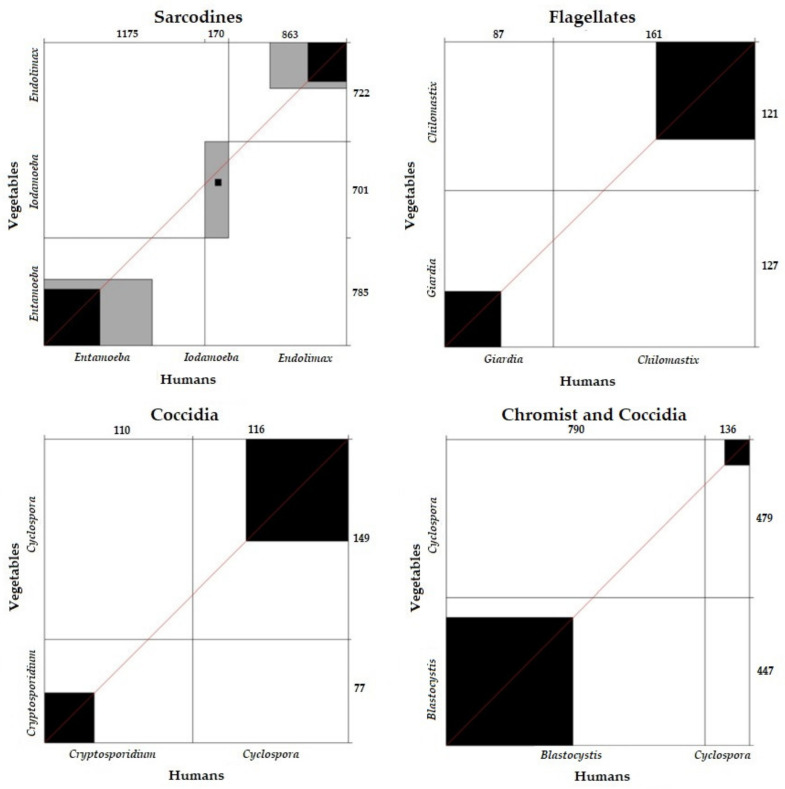
Concordance of parasite frequency between vegetables and humans according to parasite group.

Concordance analysis between the sarcodines (k = 0.35), flagellates (k = 0.27), coccidia (k = 0.28), and chromists (k = 0.37) detected in vegetables and humans was verified. The results were concordant, showing that the sarcodines, flagellates, coccidia, and chromists detected in vegetables were also diagnosed in humans (Figure 9).

##### Correlation and Concordance of Parasite Frequency between Humans and Arthropods According to Parasite Group

The comparative analysis of sarcodines (χ^2^ = 0; *p* = 1), flagellates (χ^2^ = 0.06; *p* = 0.8876), coccidia (χ^2^ = 0.024; *p* = 1), and chromists (χ^2^ = 25.14 *p* = 0.0005) in humans and arthropods showed that there were no significant differences between them. The sarcodines, flagellates, and coccidia found in arthropods and those diagnosed in humans were correlated.

**Figure 10:**
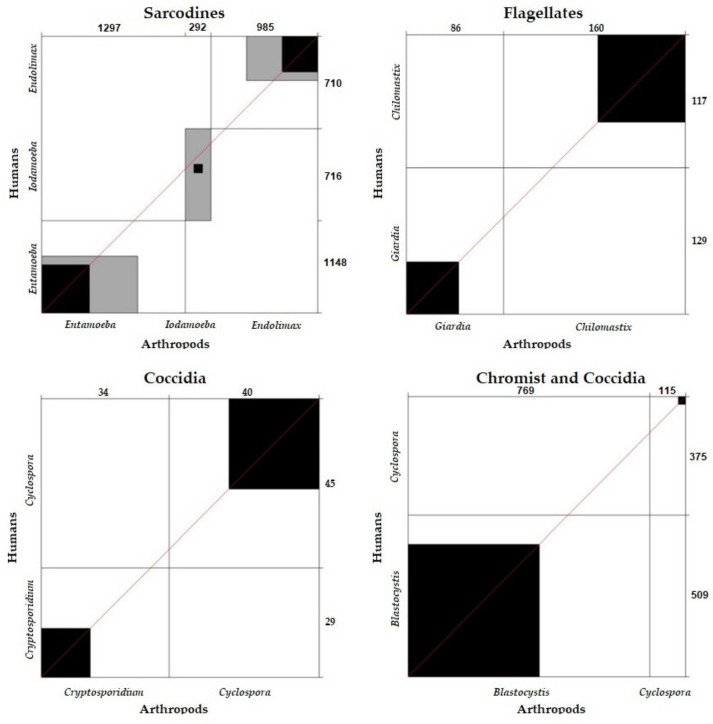
Concordance of parasite frequency between humans and arthropods according to parasite group.

Concordance analysis between the sarcodines (k = 0.36), flagellates (k = 0.27), coccidia (k = 0.27), and chromists (k = 0.40) detected in human and arthropods was verified. The results were concordant, showing that the sarcodines, flagellates, coccidia, and chromists detected in arthropods were also diagnosed in humans (Figure 10).

##### Correlation and Concordance of Parasite Frequency between Vegetables and Arthropods According to Parasite Group

The comparative analysis of sarcodines (χ^2^ = 0; *p* = 1), flagellates (χ^2^ = 0.06; *p* = 0.8876), coccidia (χ^2^ = 0.024; *p* = 1), and chromists (χ^2^ = 0.41; *p* = 0.5837) in vegetables and arthropods showed that there were no significant differences between them. The sarcodines, flagellates, coccidia, and chromists found in vegetables and those detected in arthropods were correlated.

**Figure 11:**
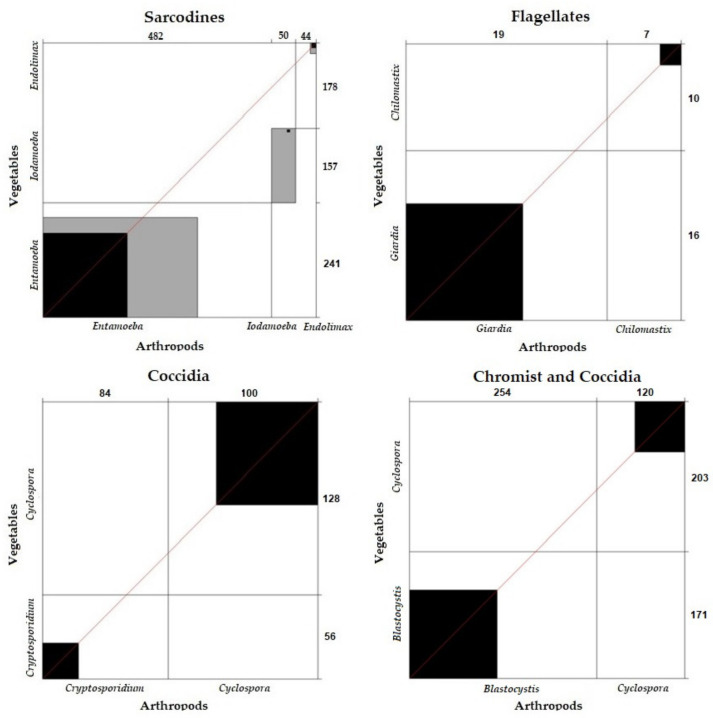
Concordance of parasite frequency between vegetables and arthropods according to parasite group.

Concordance analysis between the sarcodines (k = 0.36), flagellates (k = 0.27), coccidia (k = 0.27), and chromists (k = 0.28) detected in vegetables and arthropods was verified. The results were concordant, showing that the chromists, sarcodines, flagellates, and coccidia detected in vegetables were also detected in arthropods (Figure 11).

## 4. Discussion

The rural areas of Ecuador face a health transition that includes the improvement of environmental sanitation conditions in agricultural communities. However, deficiencies persist that must be corrected due to the continuation of health issues (diarrhea and intestinal parasites being the most common among childhood diseases) [11]. Therefore, it is necessary to carry out major works with social participation to provide feasible solutions.

It was determined that most of these risks are a consequence of the lack of hygienic–sanitary education, as confirmed by the records of the INEC [22], in which it is indicated that the San Andres parish has an illiteracy rate of 17.1% and an indigenous population of 36.9% [11,22], with residents maintaining habits and traditions, sometimes lacking adequate hygienic–sanitary measures [15]. Furthermore, poverty (88.7%) and extreme poverty (48.8%) [11] registered for this population lead to a low socioeconomic and educational level, which makes it impossible to apply preventive measures, as well as to improve the construction of houses in order to address the lack of basic sanitary infrastructure [11,14].

When analyzing the influence of environmental sanitation conditions and hygienic habits on intestinal parasitic infection in the inhabitants of the communities analyzed, we found that fecal water contamination, the absence of water tanks in the community, the absence of sanitary control in the production chain and food marketing, breeding of herbivores and pigs in the peridomicile, and dwellings with earth floors/walls and thatched roofs constituted the main risk factors in parasitic transmission. The importance of water is well known due to the resistance shown by the infective forms of parasites—especially protozoan cysts and *Blastocystis* morphotypes, which use water for their dissemination, as confirmed by González et al. [18]. In addition, the lack of construction of tanks that allow water chlorination influences transmission, in accordance with what was shown in [27]—that by implementing improvements in hygiene, with quality water, the child population recovers its growth rate, and morbidity and mortality due to diarrhea decrease. To provide an immediate solution to water problems, in these communities, the populations are trained to implement boiling treatments and adopt the water purification system known as SODIS (solar water disinfection process) [28]. In addition, work is currently being performed on the design and construction of artisanal filtration systems, made with lithological materials from Ecuador, while the competent authorities, supported by the technical reports consigned in their offices, manage the necessary resources for the construction of water treatment plants for consumption and sewage treatment [5].

The results of this study, in relation to the insufficient chlorination of the water, are consistent with those reported in indigenous Venezuelans of the state of Zulia [29], and more specifically with the study carried out in the parish of San Andres, where the contamination of all of the samples of irrigation water circulating in canals and stagnant in wells was verified [18]. These results are also in accordance with those described by Esteban et al. [30], who verified the efficient parasitic dispersal of zoonotic species through irrigation canals in Peruvian agricultural communities located at high altitudes.

Additionally, the contamination of 57% of the water samples obtained from the taps of houses has been indicated [18]—a situation that explains the considerable frequency of enteroparasitosis that the individuals of this community present, whereby almost the entire population was parasitized.

In relation to the absence of sanitary control in the production chain and food marketing, as part of the mitigation plan, the population was made aware of the need for the adequate sanitation of vegetable products obtained from these crops, and alerted about the risk of contamination inherent after washing and irrigation with contaminated water from wells and canals. The implementation of sprinkler or drip irrigation systems, the construction of supply wells far from septic tanks [18], and the periodic antiparasitic treatment of animals [19] were also suggested.

Geohelminths are not found in this Andean zone due to the extreme environmental conditions, determined by the altitude (3020–6310 m above sea level), the low temperatures (0–19 °C), and the intense solar radiation, which generates the high evapotranspiration of humidity left by the low rainfall (250 and 500 mm/year) [11]. In addition, these conditions do not allow the evolution of the soil, constituting very thin layers of lithic materials of volcanic origin [18].

Only in Community 5 was the frequency of *Ascaris lumbricoides* significantly higher than in the other communities; it is important to note that the infected people came from the coast, and had lived for more than five years in this area, thus explaining the infection with this geohelminth in a non-endemic area.

With respect to the mechanical vectors, when comparing the findings of this study with the descriptions of Adenusi and Adewoga [31], who found that 70.4% of captured flies carry parasites in Nigeria, this previous study is consistent with the results of our research, where 80.1% of the flies were contaminated with enteroparasites, as a consequence of the fertilization of crops with fresh excrement and the absence of pest control.

The correlation and concordance between the parasites detected in humans and arthropods verify that these two groups act as mechanical vectors of infective enteroparasites, elucidating some of the dispersion and transmission mechanisms that constitute risk factors in all of the agricultural communities analyzed.

Additionally, it was detected that rodents proliferate in grain and cereal deposits, where they break sacks to feed [19], representing an epidemiological risk for humans as carriers of zoonotic helminthosis [32], such as hymenolepiasis, which showed an overall frequency of 3.8%, reaching 12.3% in a community where the frequency of *Hymenolepis nana* was significantly higher. The residents reported the presence of rats inside the houses and their surroundings, which justifies this finding.

Inadequate food hygiene is an important risk factor in agricultural areas—especially in developing countries—associated with the transmission of enteroparasites through fruits and vegetables that are consumed raw or insufficiently cooked, without prior cleaning [6], as has been reported in Ethiopia with 43% [33] and Thailand with 17% [34].

The correlation and concordance between the parasites detected in humans and vegetables in this area provide evidence of the transmission of parasites through food.

Our research in the San Andres parish showed the parasitic contamination of 70.6% of the products that were grown, making this an important risk factor, due to the area being an agricultural zone, with most of its hectares dedicated to the cultivation of vegetables and fruits, which are distributed locally, regionally, nationally, and internationally, constituting a vehicle for parasites when consumed in non-endemic areas.

In the entire study, it was possible to verify that both fruits and vegetables are vehicles for the infective forms of parasites. The greater risk found in terms of the transmission of *Cyclospora* in vegetables is probably due to their contact with the soil. It is important that the coccidia oocysts are in the ground for sporulation to occur and become infective. This result is consistent with those obtained in other studies carried out in different areas [6,33,34].

Another aspect that corresponds with that reported in Ethiopia [33] is that a large number of parasitized adult individuals are asymptomatic, so they do not receive treatment, becoming important sources of infection for other people, especially when it comes to food handlers (from farmers and harvesters to those who process and serve food) [13,35].

We found that another important risk factor was the breeding of herbivores and pigs in the peridomicile. It is known that keeping animals in the home is a risk factor due to the houses not having basic sanitation conditions, and with parasitic infection in 90.3% of the animals in San Andres [19]. These results reveal the possibility of contagion to which the inhabitants of these communities are exposed, who maintain highly frequent direct contact with animals parasitized with zoonotic species. Additionally, animals may act as mechanical transmitters in the human population—especially in communities where the habit of indiscriminate open defecation exits, as explained by Rajoo et al. [36].

The correlation and concordance between the parasites detected in humans, vegetables, and arthropods verify that arthropods—especially flies—act as efficient mechanical vectors of enteroparasites, in accordance with the findings of Adenusi and Adewoga [31]. Similarly, agricultural products—especially vegetables—function as effective transmitting vehicles, being an epidemiological risk in non-endemic areas where they are marketed, as Machado-Moreira et al. argue [13].

Inadequate hygiene and the absence of basic infrastructure in the dwellings presented a high risk in the communities analyzed in this study; in this regard, it was found that dwellings with earth floors/walls and thatched roofs constitute the main risk factors for parasitic transmission. This element was also associated with the presence of animals inside the house, consistent with results reported in Malaysia [36].

We consider a limitation of the study to be not obtaining samples from all 31 communities, due to the difficulty in collecting the stool samples. Houses in these communities are remote, being in the mountains, and multiple visits are required to collect the minimum number of representative samples in each community. However, the presentation of these results was considered reasonable, due to the presence of global parasitic infection in almost all of the residents of the six communities (98.2%), varying from 96.1 to 100%, with no significant differences between them. The analysis of sanitary conditions and environmental contamination in the 31 communities did not show significant differences, which possibly suggests similar parasitic transmission among the rest of the communities.

Another limitation of this research is not having used molecular diagnostic techniques, knowing that molecular epidemiology allows the origin of parasitic species to be elucidated for suitable control.

It is essential to acquire the institutional commitment that allows successful and sustainable interventions to control the transmission of enteroparasites, which should be extended to other rural areas that present similar hygienic–sanitary deficiencies, both in Ecuador and in other developing countries. These interventions must be associated with mitigation plans to control the transmission of intestinal parasites, based on health education and environmental improvement programs prepared after identifying the risk factors.

## 5. Conclusions

The influence of environmental sanitation conditions and hygienic habits on intestinal parasitic infection in the inhabitants of the communities analyzed was found to include fecal water contamination, the absence of water tanks in the community, the absence of sanitary control in the production chain and food marketing, breeding of herbivores and pigs in the peridomicile, and dwellings with earth floors/walls and thatched roofs, constituting the main risk factors for parasitic transmission.

It was found that flies act as an important mechanical vector of infective parasitic forms, which proliferate due to the use of fresh excrement as crop fertilizer. Both fruits and vegetables produced in the area act as vehicles for parasites, being a risk factor associated with transmission due to the marketing of these agricultural products in other areas. Most of the residents of the six communities investigated in the parish of San Andres were parasitized by protozoa; fewer helminths were found, because the larvae of geohelminths cannot evolve in the soil due to the climatic conditions derived from the altitude, such as low temperatures, high levels of solar radiation, and evapotranspiration.

Consequently, as enteric parasites should still be considered an important public health issue in the analyzed agricultural communities, it is essential to educate the population in order to modify the habits that could be considered risk factors, while simultaneously improving infrastructure deficiencies to allow adequate environmental sanitation. Moreover, it is also important to modify the environment, and to influence the sociocultural and educational aspects, as well as the hygienic–sanitary practices of the population. These interventions must combine technical and socioeconomic actions that guarantee public health, including environmental sanitation in general, and the elimination of other factors that affect the dispersion and transmission of intestinal parasites, such as the fecal contamination of soil and water, the control of pests and domestic animals, and the proper hygiene of food and housing.

## Figures and Tables

**Table 1:** Distribution of the frequency of parasite contamination according to mechanical vectors.

Parasites	Flies		Spiders		Total		Dif.Prop	EE (Dif.Prop)	95%		RR	95% CI
	ns = 186		ns = 71		ns = 300 *							
	np	%	np	%	np	%			Ll	Ul		RR
*Blastocystis* sp.	75	40.3	6	8.5	81	27.0	0.3187	0.0488	0.2230	0.4144	15.9	15.2–16.5
*Entamoeba* spp.	149	80.1	6	8.5	155	51.7	0.7166	0.0441	0.6301	0.8030	9.5	8.7–10.2
*Endolimax nana*	3	1.6	0	0	3	1.0	-	-	-	-	-	-
*Iodamoeba butschlii*	5	2.7	0	0	5	1.7	-	-	-	-	-	-
*Giardia* spp.	6	3.2	0	0	6	2.0	-	-	-	-	-	-
*Cyclospora* spp.	8	4.3	0	0	8	2.7	-	-	-	-	-	-
*Eimeria* spp.	18	9.7	0	0	18	6.0	-	-	-	-	-	-
Protozoa	149	80.1	9	12.7	158	52.7	0.6743	0.0492	0.5780	0.7706	6.3	5.7–6.9
*Toxocara* spp.	2	1.1	0	0	2	0.7	-	-	-	-	-	-
Strongylida	1	0.5	0	0	1	0.3	-	-	-	-	-	-
Helminths	3	1.6	0	0	3	1.0	-	-	-	-	-	-
Total	149	80.1	9	12.7	158	52.7						

* The total number of arthropods was 300, including the 43 beetles that tested negative. np/ns: number of parasitized/number of analyzed; Dif.Prop: difference in proportions; EE (Dif.Prop): standard error of difference in proportions; CI: confidence interval; Ll: lower limit; Ul: upper limit; RR: relative risk.

**Table 2:** Distribution of the frequency of parasite contamination in fruits and vegetables.

Parasites	Fruits		Vegetables		Total		Dif.Prop	EE (Dif.Prop)	95% CI		RR	95% CI
	ns = 146		ns = 174		ns = 320							
	np	%	np	%	np	%			Ll	Ul		RR
*Blastocystis* sp.	48	32.9	44	25.3	92	28.8	0.0759	0.0510	−0.0240	0.1758	1.3001	0.96–1.65
*Entamoeba* spp.	18	12.3	28	16.1	46	14.4	−0.0376	0.0389	−0.1140	0.0387	0.7661	0.22–1.32
*Endolimax nana*	11	7.5	7	4.0	18	5.6	0.0351	0.0264	−0.0167	0.0869	1.8728	0.95–2.79
*Iodamoeba butschlii*	0	0	1	0.6	1	0.3	−0.0057	0.0057	-	-	-	-
*Giardia* spp.	0	0	5	2.9	5	1.6	−0.0287	0.0127	-	-	-	-
*Chilomastix* spp.	2	1.4	2	1.1	4	1.3	0.0022	0.0126	−0.0224	0.0268	1.1918	0.00–3.14
*Balantidium* spp.	0	0	8	4.6	8	2.5	−0.0460	0.0159	-	-	-	-
*Cryptosporidium* spp.	18	12.3	24	13.8	42	13.1	−0.0146	0.0377	−0.0886	0.0593	0.8938	0.32–1.46
*Cyclospora* spp.	32	21.9	60	34.5	92	28.8	−0.1256	0.0497	−0.2231	−0.0282	0.6356	0.26–1.00
*Cystoisospora* spp.	0	0	2	1.1	2	0.6	−0.0115	0.0081	−0.0273	0.0043	0.0000	-
*Eimeria* spp.	28	19.2	37	21.3	65	20.31	−0.0209	0.0450	−0.1090	0.0673	0.9019	0.46–1.34
Protozoa	98	67.1	120	69.0	218	68.1	−0.0184	0.0524	−0.1210	-	-	-
*Ascaris* spp.	0	0	1	0.6	1	0.3	−0.0057	0.0057	-	-	-	-
Strongylida	0	0	45	25.9	45	14.1	−0.2586	0.0332	-	-	-	-
Helminths	0	0	46	26.4	46	14.4	−0.2644	0.0334	-	-	-	-
Total	98	67.1	128	73.6	226	70.6						

np/ns: number of parasitized/number of analyzed; Dif.Prop: difference in proportions; EE (Dif.Prop): standard error of difference in proportions; CI: confidence interval; Ll: lower limit; Ul: upper limit; RR: relative risk.

**Table 3:** Frequency of intestinal parasites in residents of the San Andres parish.

Parasites	Communities of San Andrés													
	1		2		3		4		5		6		Total	
	ns = 110		ns = 122		ns = 57		ns = 32		ns = 51		ns = 24		ns = 396	
	np = 107	%	np = 122	%	np = 55	%	np = 32	%	np = 49	%	np =24	%	np = 389	%
*Blastocystis* sp.	91	82.7	103	84.4	53	93.0	29	90.6	45	88.2	22	91.7	343	86.6
*Entamoeba histolytica* *	31	28.2	39	32.0	18	31.6	12	37.5	15	29.4	9	37.5	124	31.3
*Entamoeba coli*	71	64.5	69	56.6	21	36.8	21	65.6	31	60.8	15	62.5	228	57.6
*Entamoeba hartmanni*	22	20.0	47	38.5	25	43.9	7	21.9	14	27.5	5	20.8	120	30.3
*Endolimax nana*	75	68.2	88	72.1	43	75.4	20	62.5	37	72.5	12	50.0	275	69.4
*Iodamoeba butschlii*	77	70.0	17	13.9	4	7.0	4	12.5	9	17.6	4	16.7	115	29.0
*Giardia duodenalis*	11	10.0	8	6.6	4	7.0	4	12.5	12	23.5	1	4.2	40	10.1
*Chilomastix mesnili*	27	24.5	21	17.2	8	14.0	9	28.1	10	19.6	2	8.3	77	19.4
*Retortamona intestinalis*	2	1.8	1	0.8	0	0	1	3.1	0	0	0	0	4	1.0
*Cryptosporidium* spp.	4	3.6	5	4.1	1	1.8	2	6.3	0	0	2	8.3	14	3.5
*Cyclospora cayetanesis*	2	1.8	10	8.2	3	5.3	0	0	2	3.9	0	0	17	4.3
Protozoa	107	97.3	122	100	55	96.5	32	100	49	96.1	24	100	389	98.2
*Ascaris lumbricoides*	2	1.8	4	3.3	2	3.5	0	0	8	15.7	1	4.2	17	4.3
*Trichuris trichiura*	1	0.9	2	1.6	2	3.5	0	0	0	0	1	4.2	6	1.5
Ancylostomidae **	0	0	1	0.8	1	1.8	0	0	0	0	0	0	2	0.5
*Enterobius vermicularis*	1	0.9	0	0	0	0	0	0	0	0	0	0	1	0.3
*Hymenolepis nana*	0	0	3	2.5	7	12.3	0	0	3	5.9	2	8.3	15	3.8
Helminths	4	3.6	8	6.6	9	15.8	0	0	9	17.6	3	12.5	33	8.3
Total	107	97.3	122	100	55	96.5	32	100	49	96.1	24	100	389	98.2

np/ns: number of parasitized/number of analyzed; * *Entamoeba histolytica*/*E. dispar*/*E. moshkovskii*/*E. bangladeshi*; ** Ancylostomidae: Ancylostoma duodenale/Necator americanus.

## Data Availability

The datasets used and analyzed during the current study are avail-able from the corresponding author on reasonable request.
